# Mindful Eating and Healthy Lifestyle Behaviors Among Women with and Without Regular Exercise Habits

**DOI:** 10.3390/healthcare14010067

**Published:** 2025-12-26

**Authors:** Handan Isiklar, Meral Kucuk Yetgin, Zuhal Aydan Saglam

**Affiliations:** 1 Department of Nutrition and Dietetics, Institute of Graduate Programs, Istanbul Bilgi University, Istanbul 34440, Türkiye; 2 Department of Internal Medicine, Faculty of Medicine, Yalova University, Yalova 77200, Türkiye; 3 Department of Coaching Education, Sports Health Sciences, Faculty of Sports Sciences, Marmara University, Istanbul 34815, Türkiye; 4 Department of Applied Health Science, School of Public Health, Indiana University, Bloomington, IN 47405, USA; 5 Department of Family Medicine, University of Health Sciences, Istanbul Training and Research Hospital, Istanbul 34722, Türkiye

**Keywords:** healthy lifestyle, health behavior, mindful eating, regular exercise, nutrition

## Abstract

**Background/Objectives:** Eating mindfulness and healthy lifestyle behaviors play a key role in preventing unhealthy weight gain. Understanding how these behaviors differ according to exercise habits can guide interventions targeting women’s health. This study aimed to compare healthy lifestyle behaviors and eating mindfulness between women with and without regular exercise habits. **Methods:** A cross-sectional, analytical, and descriptive study was conducted with 156 women: a Regular Exercise Group (REG, n = 68) and a Non-Exercise Group (NEG, n = 88). Data were collected through face-to-face interviews using the Mindful Eating Questionnaire (MEQ-30) and the Healthy Lifestyle Behavior Scale II (HLBS-II), along with dietary records and anthropometric measurements. **Results:** The REG scored significantly higher in eating discipline (*p* = 0.003) and in HLBS-II subscales of physical activity, nutrition, and stress management (*p* < 0.05). No significant differences were found in total MEQ scores, BMI-related nutrient intake, or other HLBS-II dimensions (*p* > 0.05). BMI values and smoking rates were lower in the REG (*p* < 0.05). Univariate logistic regression showed that BMI, eating discipline, physical activity, nutrition, stress management, and total HPLP-II scores were significantly associated with regular exercise (*p* < 0.05). In the multivariate model, BMI (OR = 1.114, 95% CI: 1.021–1.216) remained independently associated with regular exercise status. **Conclusions:** Although eating discipline was higher in the REG, overall mindful eating levels did not differ between groups. BMI were the strongest independent variables associated with regular exercise status, suggesting that while exercise supports positive lifestyle patterns, enhancing mindful eating may require additional targeted interventions.

## 1. Introduction

World Health Organization (WHO) has defined health not merely as the absence of disease or infirmity, but as a state of complete physical, mental, and social well-being [1].

The protection and promotion of health are closely related to individuals’ capacity to control behaviors that affect their own health, to take responsibility, and to develop sustainable positive habits [2,3].

Healthy lifestyle behavior is defined as the ability of individuals to regulate attitudes and behaviors that may positively or negatively affect their own health [4]. Health-promoting lifestyle behaviors consist of subdimensions such as health responsibility, physical activity, nutrition, spiritual development, interpersonal relations, and stress management, which collectively reflect a pattern of behavior that supports health [5]. The development of health-promoting lifestyle behaviors such as physical activity and exercise, healthy nutrition, and effective stress management is associated with better subjective health perception, reduced risk of non-communicable chronic diseases, and higher psychosocial/psychological well-being [6,7,8].

In the current literature, healthy nutrition is defined not only as the intake of macro- and micronutrients but also as a multidimensional phenomenon that interacts with the cognitive processes of eating behavior, emotions, and bodily signals [9,10]. Mindful eating and intuitive eating stand out as mindfulness-based approaches that encourage individuals to focus non-judgmentally on hunger–satiety cues, sensory experiences during eating, and emotional states [11,12,13]. Mindful eating shifts individuals’ dietary intake composition toward healthier choices. This relationship indicates a higher-quality, less processed, more plant-based dietary pattern. Therefore, mindful eating may be considered a mediating behavior for both the regulation of energy intake and the improvement of macronutrient balance [14].

Recent studies have reported that higher levels of mindful eating are associated with higher diet quality, lower consumption of ultra-processed foods, lower energy intake, and more favorable body weight indicators, while emotional eating is linked to negative outcomes regarding weight management, body mass index, and symptoms of eating disorders [15,16]. It has been shown that particularly among women, negative affective states such as stress, anxiety, and depressive mood are associated with emotional/unhealthy eating patterns and weight gain; emotional eating, together with depression, anxiety, and other lifestyle factors, may contribute to increases in body mass index [17,18,19].

Regular exercise not only provides physical benefits such as maintaining and increasing muscle strength, enhancing metabolism, and improving body composition, but also has the capacity to enhance psychological well-being [20,21]. In this respect, regular exercise is a fundamental health-promoting behavior that indirectly contributes to the subdimensions of stress management, spiritual development, and interpersonal relationships. Considering the relationship between emotional state and nutrition, the psychological improvements gained through exercise may also lead to more mindful and regulated eating behaviors; in other words, physical activity may support mindful eating through both physiological and cognitive pathways [22,23]. Consistent with these findings, studies examining the relationship between mindful/intuitive eating and physical activity have emphasized that women with higher eating awareness may have a more advantageous profile in terms of both diet quality and physical activity levels; however, it is noted that this relationship requires further investigation, particularly with respect to quantitative indicators such as exercise frequency and duration [24,25,26]. These findings underscore the importance of considering healthy lifestyle behaviors and eating awareness together within the framework of regular exercise habits.

Although the current literature highlights the protective effects of healthy nutrition, mindful eating, and regular physical activity on obesity, chronic diseases, and psychological well-being [27,28,29], studies comparing these variables in the context of exercise status within a health-promoting lifestyle framework remain limited. Furthermore, a recent meta-analysis examining mindfulness-based approaches reports that although some studies show positive outcomes such as reductions in caloric intake and improvements in diet quality, the overall evidence remains insufficient to support the conclusion that these approaches lead to increased exercise frequency or sustained, meaningful adherence to healthy eating behaviors [23]. Considering that women more frequently experience lifelong weight fluctuations, body image pressure, and tendencies toward emotional eating [30], understanding how regular exercise habits align with healthy lifestyle behaviors and eating awareness is important for planning both individual and community-based interventions. In this context, the present study aims to compare the health-promoting lifestyle behaviors and mindful eating of women who have been exercising regularly—at least three days a week for a minimum of three months—with those of women who have not engaged in regular exercise at any fitness facility within the past six months. Within this scope, it is hypothesized that women who exercise regularly will have higher health-promoting behavior as well as higher levels of eating awareness, compared with women who do not exercise regularly.

## 2. Materials and Methods

### 2.1. Study Design

This study was designed as a cross-sectional, analytical, and descriptive research study and was conducted among women who were members of five different fitness centers managed under a single administrative entity.

### 2.2. Participants

The study population consisted of individuals registered at five different branches of community sports centers operating under the same municipal organization in an urban district.

It included both individuals who had an established regular exercise routine and those who had recently enrolled in the sports centers and did not yet have an exercise habit. All women who met the inclusion criteria, volunteered to participate, and had a body mass index (BMI) above 25 kg/m^2^ were invited to the study. As exclusion criteria, individuals younger than 18 or older than 65 years, those participating in an intensive nutrition or exercise program and individuals with mental illnesses that could prevent accurate information sharing were not included in the study. Participants’ comorbidities, if present, were recorded and stratified into three groups based on their health status: no comorbidities, cardiometabolic diseases, and other diseases. The cardiometabolic disease group comprised individuals with controlled diabetes mellitus, hyperinsulinemia, hypertension, hyperlipidemia, and similar metabolic and cardiovascular conditions. This classification was applied in accordance with commonly used clinical categorizations in epidemiological research [31]. The other disease category included patients with hypothyroidism under medical treatment, allergic respiratory disorders, and diagnosis of previous lumbar herniae under control.

In this study, the sample size was calculated using the “Simple Random Sampling Sample Size Estimation” method, aiming to achieve a power level of 80% (1–β) at a significance level of α = 0.05. Based on this preliminary calculation, a minimum of 129 participants was required for the study to reach the targeted statistical power. The study population consisted of 70 individuals with a regular exercise habit (25.9%) and 200 individuals without a regular exercise habit (74.1%), totaling 270 individuals. Of these, 163 volunteered to participate in the study, and following the eligibility criteria, a final sample of 156 women was included. Seven individuals were excluded due to not meeting inclusion criteria or having incomplete data, ensuring the integrity of the final dataset.

Among the participants, 43.6% (n = 68) were categorized into the Regular Exercise Group (REG), defined as individuals who had been registered at the relevant gym for at least the past six months and had exercised at least three days per week for a minimum of three months. The remaining 56.4% (n = 88) were categorized into the Non-Exercise Group (NEG), consisting of newly registered individuals who had enrolled within the past month and had not attended any gym in the previous six months.

Participants were selected from among the volunteers registered at the sports centers where the study was conducted. Therefore, the sampling method is classified as convenience sampling rather than simple random sampling. The sample size estimation performed prior to the study was based on statistical power analysis and the assumptions of simple random sampling; however, the actual sample was limited to 156 individuals due to the number of volunteers who agreed to participate.

### 2.3. Data Collection

Information regarding the Participant Identification Questionnaire, the Mindful Eating Questionnaire (MEQ-30), and the Healthy Lifestyle Behavior Scale II (HLBS-II), as well as the 24-Hour Dietary Intake Record and anthropometric measurements administered to all participants through face-to-face interviews, is presented below.

Data were collected between December 2018 and January 2019. All data were gathered by the principal researcher through face-to-face, individual interviews conducted by appointment at the specific branch of the sports center where the participants were registered, using paper-based questionnaires.

#### 2.3.1. Participant Identification Questionnaire

The Participant Identification Questionnaire consisted of four main sections aimed at obtaining Demographic Characteristics and General Health Information. In the first section, sociodemographic characteristics (age, educational status, occupation) were assessed. The second section included anthropometric information (body weight three months ago, current body weight, height, and body mass index). The third section inquired about health information (whether the participant had any physician-diagnosed disease, whether they had previously consulted a dietitian, and their smoking and alcohol consumption habits). In the fourth section, participants were asked whether they had joined the gym voluntarily and whether their current dietary habits were being monitored.

#### 2.3.2. Mindful Eating Questionnaire (MEQ-30)

The Mindful Eating Questionnaire (MEQ), originally developed by Framson et al. (2009) as a 28-item self-report scale to assess mindful eating, eating attitudes, and the relationships between awareness and emotional states, was adapted into Turkish as the Yeme Farkındalığı Ölçeği (YFÖ-30) by Köse et al. (2016) after validity and reliability analyses [32,33].

In the Turkish version (YFÖ-30), five items were directly taken from the original scale, while the remaining items were adapted based on the same scale, forming a total of 30 questions. In the scoring system, items 1, 7, 9, 11, 13, 15, 18, 24, 25, and 27 are scored directly, whereas the remaining items are reverse-scored (Reverse Scoring: 1 = 5, 2 = 4, 3 = 3, 4 = 2, 5 = 1). Participants with higher scores are classified as having greater awareness of their hunger and satiety states, while those with lower scores are considered to have lower awareness.

The items in the scale are evaluated using a 5-point Likert scale (1: never, 2: rarely, 3: sometimes, 4: often, 5: always). The subdimensions of the scale are categorized into seven factors: disinhibition, emotional eating, eating control, focusing, eating discipline, mindfulness, and interference [33].

#### 2.3.3. Health-Promoting Lifestyle Profile II (HPLP-II)

The Health-Promoting Lifestyle Profile (HPLP) scale, originally developed by Walker et al. (1987) to measure health-promoting behaviors [34]. was later revised and renamed as HPLP-II in 1996 [35]. The Turkish validity and reliability study of the scale was conducted by Bahar et al. (2008) [36] under the title Sağlıklı Yaşam Biçimi Davranışları Ölçeği II (SYBDÖ-II). The Cronbach’s alpha reliability coefficient of the scale ranges between 0.79 and 0.94.

The scale consists of 52 items evaluated on a 4-point Likert scale (1: never, 2: sometimes, 3: often, 4: regularly). It includes six subdimensions: self-actualization, health responsibility, exercise habits, nutrition habits, interpersonal support, and stress management. The number of items and the minimum–maximum possible scores for each subdimension are as follows: spiritual growth—13 items (min 13–max 52 points), health responsibility—10 items (min 10–max 40 points), physical activity—5 items (min 5–max 20 points), nutrition—6 items (min 6–max 24 points), interpersonal relations—7 items (min 7–max 28 points), and stress management—7 items (min 7–max 28 points).

The total possible score of the scale ranges from a minimum of 52 to a maximum of 208. Higher scores indicate that individuals perform health-promoting behaviors at a better level [36].

#### 2.3.4. Dietary Intake Record

Information regarding the types and amounts of foods consumed by the participants was obtained through a 24 h dietary intake record form, completed face-to-face with the aid of the Food and Meal Catalogue [37]. The data collected from these forms were analyzed using the Nutrition Information System (BeBiS, Istanbul, Türkiye) version 7.0 software to calculate the daily energy and macronutrient intakes. BeBiS is a widely used program in nutritional studies that provides detailed information on the macro- and micronutrient contents of consumed foods.

The nutrient intake values calculated using this program were evaluated according to the Dietary Reference Intakes (DRI) based on gender and age [38]. Participants whose intake met ≤67% of the reference values were classified as insufficient, those meeting between 67% and 133% were classified as adequate, and those meeting ≥ 133% were classified as excessive intake [39].

#### 2.3.5. Anthropometric Measurements

Body height was measured using a stadiometer (Leicester, SECA GmbH & Co. KG, Hamburg, Germany) in accordance with standard procedures, with a margin of error of ±1 mm, while body weight was measured using a digital scale (Felix Form FL593, Felix, Istanbul, Türkiye) with an accuracy of ±100 g. The Body Mass Index (BMI) of the participants was calculated by dividing body weight (kg) by the square of body height (m) using the formula: BMI = Body weight (kg)/Body Height^2^ (m^2^).

BMI values were then classified according to the World Health Organization (WHO) standards. Specifically, a BMI of less than 18.5 kg/m^2^ was categorized as underweight; 18.5–24.9 kg/m^2^ as normal weight; 25.0–29.9 kg/m^2^ as overweight (pre-obese); 30.0–34.9 kg/m^2^ as Obesity Class I; 35.0–39.9 kg/m^2^ as Obesity Class II; and ≥40.0 kg/m^2^ as Obesity Class III [40].

### 2.4. Statistical Analysis

Statistical analyses were performed using the NCSS (Number Cruncher Statistical System) 2007 software (Kaysville, UT, USA). Descriptive statistical methods (mean, standard deviation, median, frequency, percentage, minimum, and maximum) were used in the evaluation of the data. The normality of distribution for quantitative variables was assessed using the Shapiro–Wilk test and graphical methods.

For comparisons between two groups involving normally distributed quantitative variables, Student’s *t*-test was applied. For non-normally distributed quantitative variables, the Mann–Whitney U test was used for two-group comparisons. In cases where non-normally distributed quantitative variables were compared across three or more groups, the Kruskal–Wallis test was employed.

Relationships between quantitative variables were examined using Pearson correlation analysis. For comparisons involving qualitative variables, the Pearson Chi-Square test, Fisher-Freeman-Halton test, were utilized.

To examine the association between regular exercise status and potential explanatory variables, univariate logistic regression analyses were initially performed, followed by a multivariate logistic regression model to control for potential confounders (age, BMI, and presence of disease), and odds ratios (ORs) with 95% confidence intervals (CIs) were calculated.

Cohen’s d coefficient was used to assess mean differences between continuous variables; d = 0.20 was interpreted as a small effect, d = 0.50 as a medium effect, and d = 0.80 or higher as a large effect. For relationships between categorical variables, the Phi (φ) coefficient was calculated for 2 × 2 tables, and Cramér’s V was used for larger contingency tables. Phi and Cramér’s V values were evaluated according to effect size thresholds of 0.10 for small, 0.30 for medium, and 0.50 for large effects. The interpretation of effect sizes was based on Cohen’s recommended classifications [41].

Statistical significance was considered at a level of *p* < 0.05.

## 3. Results

Table 1 compares the groups in terms of demographic, anthropometric, nutritional, and health-related characteristics. The BMI values of REG were found to be significantly lower than those of NEG (*p* = 0.013). Similarly, a statistically significant difference was observed between the groups regarding smoking status; the rate of smoking was lower in REG compared to NEG (*p* = 0.048). In terms of sources followed for current nutrition information, the proportion of obtaining information from television and the internet was higher in NEG than in REG (*p* = 0.003). Regarding the number of daily snacks consumed, REG reported a significantly higher number of snacks compared to NEG (*p* = 0.013). No statistically significant differences were observed between the groups concerning following current nutrition information, previous visits to a dietitian, or daily snack consumption status (*p* > 0.05).

The difference between the mean BMI of the regular exercise group three months prior (30.70 ± 4.38) and their current mean BMI (29.54 ± 3.56) was found to be statistically significant (*p* < 0.001). This finding indicates the effect of three months of exercise participation on weight management.

A statistically significant difference was also observed between the groups in terms of the number of daily snacks consumed, with the regular exercise group reporting a higher number of snacks compared to the non-exercise group (*p* = 0.013).

Table 2 presents the comparison of Mindful Eating Questionnaire (MEQ-30) scores between the groups. Accordingly, no statistically significant differences were observed between the groups in terms of emotional eating, eating control, focus, awareness, interference, or total scores (*p* > 0.05). Although the difference in disinhibition scores was not statistically significant (*p* = 0.092), the higher disinhibition scores observed in REG are noteworthy. On the other hand, a statistically significant difference was found between the groups in terms of eating discipline scores, with REG showing higher scores than NEG (*p* = 0.003).

Table 3 presents the comparison of Health-Promoting Lifestyle Profile II (HPLP-II) scores between the groups. According to the intergroup comparisons, the total HPLP-II score of REG was significantly higher than that of NEG (*p* = 0.001). Similarly, REG demonstrated significantly higher scores than NEG in the subscales of physical activity (*p* = 0.001), nutrition (*p* = 0.015), and stress management (*p* = 0.006). Although no statistically significant differences were observed between the groups in the subscales of health responsibility (*p* = 0.054) and spiritual growth (*p* = 0.074), the scores of REG were notably close to statistical significance and higher than those of NEG. No significant difference was found between the groups in terms of interpersonal relations (*p* > 0.05).

Table 4 presents the evaluation of the ratios of meeting the recommended values for energy, carbohydrate, protein, and fat intakes according to the DRI reference across the groups. No statistically significant differences were found between the regular exercise and non-exercise groups in terms of the amounts of energy, carbohydrate, protein, and fat intake recommended by the DRI (*p* > 0.05).

In the univariate logistic regression analysis, current BMI, eating discipline, physical activity, nutrition, stress management, and the total HPLP-II score were found to be statistically significantly associated with regular exercise status (*p* < 0.05). As BMI increases, the likelihood of not engaging in regular exercise increases (OR: 1.094, 95% CI: 1.012–1.182, *p* = 0.024). An increase in eating discipline decreases the likelihood of not engaging in regular exercise (OR: 0.866, 95% CI: 0.784–0.956, *p* = 0.004). Similarly, increases in physical activity (OR: 0.864, 95% CI: 0.809–0.922, *p* < 0.001), nutrition (OR: 0.909, 95% CI: 0.842–0.982, *p* = 0.016), stress management (OR: 0.896, 95% CI: 0.826–0.972, *p* = 0.008), and the total HPLP-II score (OR: 0.975, 95% CI: 0.959–0.990, *p* = 0.002) were associated with a decreased likelihood of not engaging in regular exercise. In contrast, age and the presence of disease were not statistically significant in the univariate analyses (*p* > 0.05) (Table 5).

In the multivariate logistic regression analysis conducted to control for the effects of potential confounders (age, BMI, and the presence of disease), only the physical activity variable remained independently associated with regular exercise (OR = 0.862; 95% CI: 0.788–0.943; *p* = 0.001). Current BMI also retained statistical significance in the multivariate model, indicating that an increase in BMI was associated with a higher likelihood of not engaging in regular exercise (OR = 1.114; 95% CI: 1.021–1.216; *p* = 0.016) (Table 5).

In the multivariate analysis, age, presence of disease, eating discipline, nutrition, stress management, and the total HPLP-II score lost their significance. This suggests that the associations observed in the univariate analyses can largely be explained by the effects of confounding factors (Supplementary Figure S1).

## 4. Discussion

The most salient finding of our study, which explored the relationship between mindful eating and health-promoting lifestyle behaviors among individuals with and without regular exercise habits, is that stress management scores—an essential determinant of quality of life—were significantly higher in the group engaging in regular exercise. This was observed alongside modifiable components of health-promoting behaviors, namely physical activity and nutrition. Additionally, the higher eating discipline scores identified in the regularly exercising group suggest that certain dimensions of dietary behavior may be more favorable among individuals who maintain an exercise routine. Nevertheless, regular exercise did not yield a statistically significant difference in overall mindful eating levels. Furthermore, our findings indicate that the association between body mass index and regular exercise behavior appears to be independent of other lifestyle components, underscoring the potential role of weight status as a key factor in shaping exercise habits.

### 4.1. Differences in Baseline Characteristics

Our study demonstrates that the BMI values of the regular exercise group (REG) were significantly lower compared to the non-exercise group (NEG), and that an additional reduction in BMI was observed over the three-month exercise period (from 30.70 to 29.54, *p* = 0.0002), supporting the association between regular exercise and weight management. Previous research identifies regular exercise as an independent modality in the treatment of overweight and obesity, emphasizing its beneficial effects on weight loss, cardiorespiratory fitness, metabolic markers, and long-term weight maintenance [42,43].

Although the relationship between exercise and body composition in our study was assessed solely through BMI, this approach aligns with guidelines that consider exercise a fundamental component of weight management [44]. In our study, despite the higher daily snacking frequency observed in the REG compared to the NEG, BMI remained lower, which is consistent with research examining the “eating frequency–body weight” relationship. The inverse association between snacking frequency and BMI appears to be particularly pronounced among individuals with higher levels of physical activity [45].

Indeed, physical activity has been shown to enhance appetite-regulating mechanisms, promoting a better alignment between energy intake and expenditure, with more active individuals demonstrating superior compensatory responses to meals of varying energy content [46]. A recent cohort study also indicated that changes in smoking status may be associated with adverse changes in diet and physical activity habits, suggesting that smoking cessation or abstinence tends to co-occur with healthier lifestyle patterns [47]. In this context, the lower prevalence of smoking observed in the REG may reflect that participation in exercise fosters an environment conducive to smoking cessation through heightened awareness, increased health motivation, and social interaction channels among individuals with similar health-oriented goals.

Although the NEG used television and the internet more extensively, their disadvantage in terms of BMI may be explained by the quality of media-based nutritional information and the association between increased screen time and sedentary behavior [48].

### 4.2. Mindful Eating and Exercise Habit

In the previous literature, only a limited number of studies have examined the concept of mindfulness, mindful eating behaviors, and their relationship with exercise. Due to the inconsistency of findings, these studies have not been able to establish a direct link or reach definitive conclusions regarding the association of mindfulness and mindful eating on exercise behavior [49,50,51]. In fact, one study found that more physically active students were less likely to be aware of their food choices and exhibited a higher tendency toward emotional eating [52]. Conversely, another study revealed that individuals with lower levels of physical activity demonstrated an increased tendency toward emotional eating [53]. Contrary to these findings, a study conducted by Mert-Biberoğlu et al. (2024) reported that adequate and balanced nutrition combined with physical activity supports intuitive and mindful eating behaviors in young individuals, thereby contributing to their overall health [54]. However, a separate study determined that there was no relationship between the mindful eating levels of young individuals who spend their leisure time engaging in physical activity [55].

In our study, exercise did not appear to make a significant difference in the subdimensions of mindful eating, namely emotional eating, eating control, focusing, awareness, and interference disinhibition. The only notable finding was that eating discipline was higher among the group that engaged in regular exercise. Although previous literature has demonstrated significant relationships between body image and emotional eating, our findings suggest that exercise still does not have a distinct association on the subdimensions of mindful eating.

However, there are studies supporting the view that as mindful eating behavior increases, healthier food choices are made, leading to a decrease in BMI [56,57]. In our study, although there was no significant difference in calorie intake and mindful eating between the group that exercised regularly and the group that did not, the decrease in BMI rates among the regularly exercising group can be explained by the direct increase in energy expenditure due to exercise, rather than a reduction in food consumption.

However, the fact that exercise enhances eating discipline suggests that it may indirectly support healthier eating behaviors. The decrease in BMI observed in the group that engaged in regular exercise may have led to increased body awareness, which in turn could result in higher self-control, a tendency to make healthier food choices, and greater resistance to unhealthy foods. For instance, it has been reported that individuals with higher levels of mindfulness tend to consume energy-dense foods in smaller portions [22].

### 4.3. Health-Promoting Lifestyle Profile and Exercise Habit

Although the benefits of exercise in weight management were observed in our study, these association were not sufficiently reflected in mindful eating. Nevertheless, positive implications for healthy lifestyle behaviors were evident. When comparing the groups, the subdimensions of healthy lifestyle behavior, namely physical activity, nutrition, and stress management, were found to be statistically significant in favor of the group that engaged in regular exercise, which was also reflected in the overall endorphin release due to exercise, which reduces stress and anxiety, thereby decreasing tendencies toward emotional eating. Consequently, individuals may have used exercise as a coping mechanism instead of turning to food in response to challenging emotions.

A recent study conducted on women revealed that an eight-week healthy lifestyle behavior intervention led to a reduction in emotional and external eating behaviors compared to the control group, and that the women in the intervention group lost an average of 1.5 kg more weight. The study also confirmed that participants with higher levels of stress exhibited greater tendencies toward emotional and external eating behaviors and were more likely to be overweight [58]. In women, both the hypothalamic–pituitary–adrenal axis response and reward system activation facilitate a preference for high-calorie, sugary, and fatty foods under stress, leading to increased levels of emotional eating during periods of intense stress [59]. Therefore, gender differences should be taken into account when designing lifestyle interventions focused on stress management and emotional eating.

Stress can influence behavior by triggering overeating and the consumption of high-calorie, fatty, or sugary foods; by reducing physical activity; and by shortening sleep duration. The results of two cross-sectional studies conducted by Leipold and colleagues demonstrate that physical activity and nutritional awareness serve as important protective factors for resilience by buffering the relationship between life satisfaction and stress. Moreover, the studies indicate that stress negatively affects healthy dietary choices [60]. In our study, the higher levels of awareness regarding exercise and nutrition, as well as the ability to manage stress observed in the group that engaged in regular exercise, support Leipold’s finding that a healthy lifestyle functions as a protective factor against stress.

On the other hand, different levels of physical activity have not been found to have a significant effect on mindful eating [61]. It has been observed that individuals who practice yoga exhibit more favorable eating behaviors, higher quality of life, greater satisfaction with body image, healthier body weight, and better emotional regulation in response to stress [62]. These findings suggest that further research is needed to explore the association of various exercise modalities and intensities on mindful eating and healthy lifestyle behaviors.

### 4.4. Nutrient Intake and Exercise Habit

When the dietary intake of the groups was compared with the Dietary Reference Intakes (DRI), it was observed that energy intake in both groups was significantly below the recommended levels (REG: 46.88%; NEG: 42.52%), with approximately 87% of participants exhibiting insufficient intake at or below 67% of the recommended level. Similarly, carbohydrate intake was low, with group averages falling below the recommended range and nearly half of the participants showing inadequate intake. Although protein intake was within the recommended limits on average, the rate of inadequacy was notable, particularly in the NEG (52.3%). Fat intake, on the other hand, was found to be high in both groups, with the majority of participants (>64%) falling into the excessive intake category.

Although regular exercise slightly increased protein intake and marginally raised energy intake, it did not significantly improve the overall macronutrient balance. Both exercising and non-exercising individuals were characterized by low energy and carbohydrate intake, and the issue of high fat consumption persisted across both groups.

Lieberman et al. (2020) reported that physical activity does not consistently affect the percentage of energy derived from protein or fat, and that in some populations, the proportion of protein remains stable while the distribution of carbohydrates and fats varies [63]. This finding aligns with our study, in which the average protein intake was adequate, yet the percentage of fat intake remained high. Numerous studies have also reported that exercise alone does not consistently alter energy or macronutrient intake [64]. This finding may explain why the deficiencies in energy and carbohydrate intake observed in our study were not automatically corrected through exercise. While individuals who engage in regular exercise may have a relative advantage in protein intake, exercise alone may not be sufficient to correct poor macronutrient distribution, such as energy and carbohydrate deficiencies and excessive fat intake [65]. Our findings indicate that exercise alone does not improve dietary quality and suggest that it should be supported by balanced nutritional interventions.

### 4.5. Limitations

This study has several limitations. First, its cross-sectional design prevents causal inference, and the findings should be interpreted as associations only. The sample consisted exclusively of women attending sports centers affiliated with a single institution, which may introduce selection bias and limits generalizability. The proportion of regular exercisers was also higher than that observed in the general population, further restricting representativeness. Although simple random sampling was intended, the lack of a complete sampling frame resulted in a sampling process closer to convenience sampling, which may limit the ability of the sample to fully represent the target population. Self-reported data may be affected by recall or social desirability bias. Dietary intake was assessed using a single 24 h recall; because a second contact was not possible, the preferred three-day recall method could not be implemented, limiting the ability to capture day-to-day variability. Additionally, only BMI was measured, and body composition indicators such as fat and lean mass were not assessed. In multivariate analysis, some variables lost significance compared to univariate results, likely reflecting shared variance among lifestyle-related factors rather than independent associations. Finally, because the study included only women, the results cannot be generalized to men or to broader populations. Future research with larger, more diverse samples and longitudinal designs is needed to better assess causality and the direction of lifestyle behavior changes.

### 4.6. Future Recommendations

Future studies should explore how different exercise modalities, frequencies, and intensities are associated with mindful eating and health-promoting lifestyle behaviors, particularly using longitudinal designs that allow assessment of behavioral change over time. Investigating body composition indicators and employing more detailed dietary assessment methods may provide deeper insight into the mechanisms underlying these associations. Integrating mindfulness-based nutrition education into exercise programs and promoting community initiatives that combine physical activity with eating awareness may support more sustainable lifestyle changes in women. Additionally, clinicians should consider incorporating mindfulness-based strategies into routine nutrition and physical activity counseling to enhance patient engagement and adherence, provide personalized exercise prescriptions that encourage mindful eating habits, and monitor behavioral changes over time through follow-up assessments. Collaboration with community programs that combine exercise with mindful eating education and offering education on body composition metrics and diet quality can further support holistic and sustainable lifestyle modifications.

## 5. Conclusions

In this cross-sectional study, women who reported engaging in regular exercise showed higher scores in the physical activity, nutrition, and stress management subdimensions of the HLBS-II, as well as higher eating discipline scores, compared with women who did not exercise regularly. However, no significant differences were observed between groups in total mindful eating scores or BMI-related nutrient intake.

Logistic regression analyses indicated that several lifestyle-related variables, including physical activity, nutrition, stress management, and eating discipline, were associated with regular exercise status in univariate models. In the multivariate analysis, BMI remained the only independent variable significantly associated with regular exercise status.

These findings suggest that while regular exercise is linked to more favorable health-promoting lifestyle behaviors, it may not be sufficient on its own to improve overall mindful eating. Given the cross-sectional design of the study, causal relationships cannot be inferred, and future longitudinal or interventional studies are warranted to clarify the directionality of these associations and to determine whether targeted strategies are needed to enhance mindful eating behaviors alongside exercise interventions.

## Figures and Tables

**Table 1:** Comparison of demographic, anthropometric, nutritional, and health-related characteristics (n = 156).

		REG (n = 68)	NEG (n = 88)	*p*	Effect Size
Age (years)	$\bar{X}$ ± SD	40.57 ± 9.80	37.91 ± 9.53	^a^ 0.089	^x^ 0.276
Height (cm)	$\bar{X}$ ± SD	159.79 ± 5.48	160.72 ± 5.80	^a^ 0.315	^x^ 0.163
Body Weight 3 Months Ago (kg)	$\bar{X}$ ± SD	78.31 ± 11.13	80.64 ± 14.65	^a^ 0.278	^x^ 0.176
Current Body Weight (kg)	$\bar{X}$ ± SD	75.44 ± 9.90	80.60 ± 13.78	^a^ 0.007	^x^ 0.421
Current BMI (kg/m^2^)	$\bar{X}$ ± SD	29.76 ± 3.60	31.62 ± 5.65	^a^ 0.013	^x^ 0.404
Presence of Comorbidities	Yes	25 (36.8)	35 (39.8)	^b^ 0.702	^φ^ 0.031
	No	43 (63.2)	53 (60.2)		
	Cardiometabolic Diseases	16 (61.5)	23 (65.7)	^b^ 0.945	^φ^ 0.04
	Other	12 (46.2)	16 (45.7)		
Status of Following Current Nutrition Information	Yes	32(47.1)	38(43.2)	^b^ 0.629	^φ^ 0.027
	No	36 (52.9)	50 (56.8)		
Sources Used to Follow Current Nutrition Information	TV	15 (46.9)	10 (26.3)	^c^ 0.003	^v^ 0.430
	Internet	7 (21.9)	11 (28.9)		
	TV + Internet	5 (15.6)	17 (44.7)		
	Dietitian	5 (15.6)	0 (0)		
History of Visiting a Dietitian	Yes	34 (50.0)	40 (45.5)	^b^ 0.573	^φ^ 0.045
	No	34 (50.0)	48 (54.5)		
Smoking Status	Smokers; n(%)	3 (4.4)	12 (13.6)	^b^ 0.048	^φ^ 0.155
	Non-smokers; n(%)	65 (95.6)	76 (86.4)		
Number of Main Meals per Day	Min-Max (Median)	1–5 (2)	1–3 (2.5)	^d^ 0.756	^x^ 0.039
Mid Meal Snack Consumption Status	Yes	59 (86.8)	71 (80.7)	^b^ 0.312	^φ^ 0.080
	No	9 (13.2)	17 (19.3)		
Number of Mid Meal Snacks per Day	Min-Max (Median)	1–4 (2)	1–3 (1)	^d^ 0.013	^x^ 0.406

REG: Regular Exercise Group, NEG: No Exercise Group; ^a^: Student’s t-Test; ^b^: Pearson Chi-Square Test; ^c^: Fisher–Freeman–Halton Test; ^d^: Mann–Whitney U Test; ^x^: Cohen’s d, ^φ^: Phi, ^v^: Cramer’s V.

**Table 2:** Comparison of Mindful Eating Questionnaire (MEQ-30) scores (n = 156).

	REG (n = 68) $\bar{X}$ ± SD	NEG (n = 88) $\bar{X}$ ± SD	^a^ *p*	Cohen’s d
Disinhibition	16.59 ± 4.12	15.32 ± 5.00	0.092	0.274
Emotional eating	16.28 ± 5.24	16.41 ± 5.96	0.887	0.023
Eating control	14.37 ± 4.37	13.76 ± 4.86	0.421	0.130
Focusing	17.18 ± 2.27	16.95 ± 2.37	0.556	0.020
Eating discipline	12.43 ± 3.50	10.82 ± 3.21	0.003	0.481
Awareness	15.91 ± 3.30	15.70 ± 3.43	0.705	0.061
Interference	7.66 ± 1.79	7.20 ± 1.86	0.124	0.212
Total	100.41 ± 14.18	96.17 ± 18.49	0.107	0.248

^a^ Student *t*-test; Effect size: Cohen’s d.

**Table 3:** Comparison of Health-Promoting Lifestyle Profile II (HPLP-II) scores (n = 156).

	REG (n = 68) $\bar{X}$ ± SD	NEG (n = 88) $\bar{X}$ ± SD	^a^ *p*	Cohen’s d
Health responsibility	25.81 ± 5.70	23.92 ± 6.27	0.054	0.320
Physical activity	21.85 ± 4.74	17.61 ± 5.68	0.001	0.804
Nutrition	23.60 ± 4.27	21.91 ± 4.26	0.015	0.404
Spiritual growth	28.82 ± 4.22	27.49 ± 4.87	0.074	0.325
Interpersonal relations	28.56 ± 4.51	28.41 ± 4.44	0.836	0.015
Stress management	21.51 ± 3.85	19.68 ± 4.23	0.006	0.446
Total	150.16 ± 20.52	139.02 ± 20.95	0.001	0.546

^a^ Student *t*-test; Effect size: Cohen’s d.

**Table 4:** Comparison of the ratios of meeting the recommended values for energy and macronutrient intakes according to the DRI.

		REG (n = 68)	NEG (n = 88)	*p*	Effect Size
Consumed Energy (kcal/day)	$\bar{X}$ ± SD	1054.77 ± 796.48	964.42 ± 493.63	^a^ 0.386	^x^ 0.140
Required energy intake (kcal/day)	$\bar{X}$ ± SD	2252.4 ± 67.64	2270.16 ± 65.82	^a^ 0.101	^x^ 0.267
Percentage of energy intake meeting (%)	$\bar{X}$ ± SD	46.88 ± 35.64	42.52 ± 21.59	^a^ 0.346	^x^ 0.153
Energy intake level (n, %)	Insufficient	59 (86.8)	77 (87.5)	^c^ 0.694	^v^ 0.090
	Adequate	8 (11.8)	11 (12.5)		
	Excessive intake	1 (1.5)	0 (0)		
Carbohydrate intake (gr)	$\bar{X}$ ± SD	97.23 ± 53.65	104.71 ± 68.05	^a^ 0.457	^x^ 0.120
Carbohydrate intake (%)	$\bar{X}$ ± SD	39.50 ± 14.28	43.49 ± 15.33	^a^ 0.099	^x^ 0.268
Carbohydrate intake level (n, %)	Insufficient	34 (50)	41 (46.6)	^a^ 0.595	^v^ 0.080
	Adequate	27 (39.7)	33 (37.5)		
	Excessive intake	7 (10.3)	14 (15.9)		
Protein intake (gr)	$\bar{X}$ ± SD	38.66 ± 20.48	33.87 ± 18.99	^a^ 0.133	^x^ 0.244
Protein intake (%)	$\bar{X}$ ± SD	16.07 ± 5.89	15.27 ± 6.36	^a^ 0.422	^x^ 0.130
Protein intake level (n, %)	Insufficient	26 (38.2)	46 (52.3)	^b^ 0.211	^v^ 0.140
	Adequate	34 (50)	33 (37.5)		
	Excessive intake	8 (11.8)	9 (10.2)		
Fat intake (g)	$\bar{X}$ ± SD	55.82 ± 75.09	44.72 ± 27.42	^a^ 0.202	^x^ 0.207
Fat percentage (%)	$\bar{X}$ ± SD	44.31 ± 14.43	41.28 ± 14.48	^a^ 0.197	^x^ 0.210
Fat intake level (n, %)	Insufficient	3 (4.4)	6 (6.8)	^c^ 0.731	^v^ 0.070
	Adequate	17 (25)	25 (28.4)		
	Excessive intake	48 (70.6)	57 (64.8)		

REG: Regular Exercise Group, NEG: No Exercise Group; DRI: Dietary Reference Intakes; ^a^: Student *t*-test; ^b^: Pearson Chi-Square Test; ^c^: Fisher Freeman Halton Test; ^x^: Cohen’s d, ^v^: Cramer’s V.

**Table 5:** Univariate and Multivariate Logistic Regression Analysis Results.

	Univariate		Multivariate	
	OR (%95 CI)	*p*	OR (%95 CI)	*p*
Age (years)	0.972 (0.940–1.005)	0.091	0.967 (0.929–1.007)	0.100
BMI (kg/m^2^)	1.094 (1.012–1.182)	**0.024**	1.114 (1.021–1.216)	**0.016**
Presence of comorbidities	1.136 (0.592–2.180)	0.702	1.168 (0.547–2.492)	0.689
Eating discipline	0.866 (0.784–0.956)	**0.004**	0.950 (0.844–1.068)	0.388
Physical activity	0.864 (0.809–0.922)	**<0.001**	0.862 (0.788–0.943)	**0.001**
Nutrition	0.909 (0.842–0.982)	**0.016**	0.976 (0.864–1.102)	0.697
Stress management	0.896 (0.826–0.972)	**0.008**	0.961 (0.844–1.095)	0.551
HPLP-II Total	0.975 (0.959–0.990)	**0.002**	1.011 (0.976–1.047)	0.535

OR: Odds Ratio, CI: Confidence Interval, The multivariate model was adjusted for age, BMI, and presence of disease. Bold values indicate statistically significant results (*p* < 0.05).

## Data Availability

The data that support the findings of this study are available from the corresponding author upon reasonable request.

## Supplementary Materials

The following supporting information can be downloaded at: https://www.mdpi.com/article/10.3390/healthcare14010067/s1, Figure S1: Logistic regression results for estimated odds ratios and 95% confidence intervals of the multivariate model by groups, (adjusted for age, BMI, and presence of disease).
