# Supplementary material for: Mindful Eating and Healthy Lifestyle Behaviors Among Women with and Without Regular Exercise Habits
Source: Healthcare (Basel). 2025 Dec 26;14(1):67. doi: 10.3390/healthcare14010067 (PMC12785492; doi:10.3390/healthcare14010067)
Supplement: Supplementary file 1 [file healthcare-14-00067-s001.zip › healthcare-4029169-supplementary.pdf]

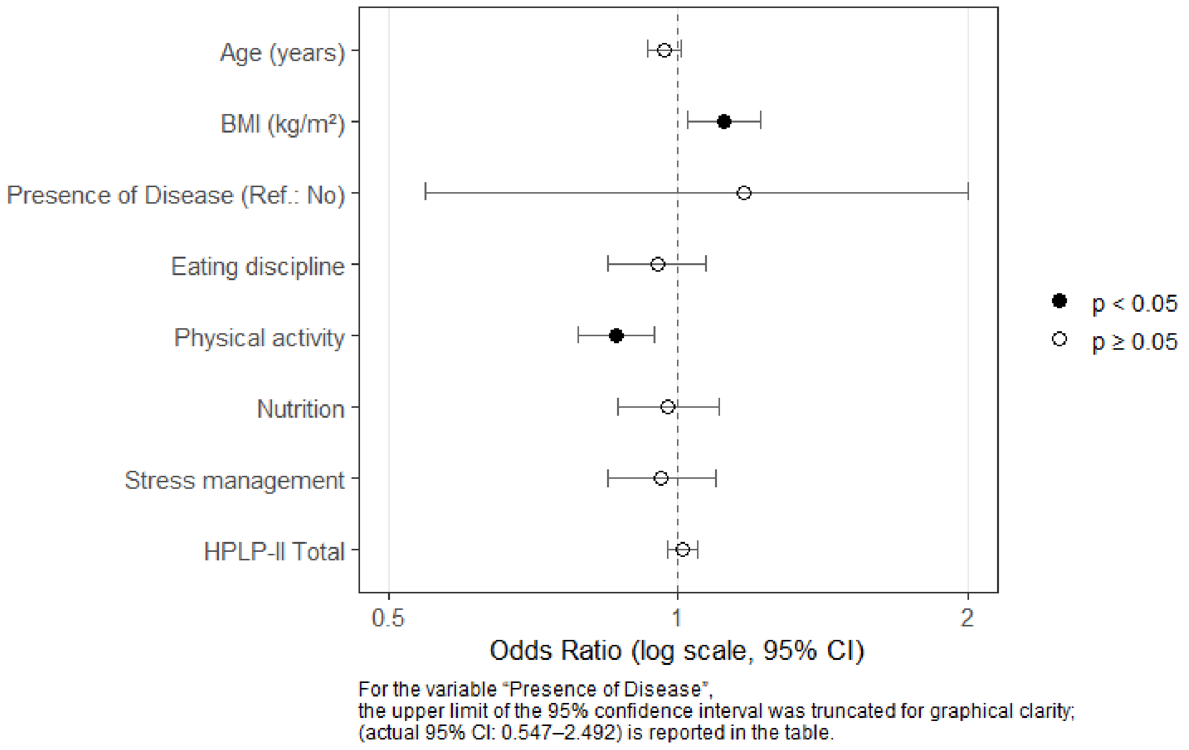

Figure S1. Logistic regression results for estimated odds ratios and 95% confidence in-tervals of the multivariate model by groups, (adjusted for age, BMI, and presence of disease).
